# Soft Transducer for Patient’s Vitals Telemonitoring with Deep Learning-Based Personalized Anomaly Detection

**DOI:** 10.3390/s22020536

**Published:** 2022-01-11

**Authors:** Pasquale Arpaia, Federica Crauso, Egidio De Benedetto, Luigi Duraccio, Giovanni Improta, Francesco Serino

**Affiliations:** 1 Interdepartmental Research Center in Health Management and Innovation in Healthcare (CIRMIS), University of Naples Federico II, 80125 Naples, Italy; pasquale.arpaia@unina.it; 2 Department of Information Technology and Electrical Engineering (DIETI), University of Naples Federico II, 80125 Naples, Italy; 3 Department of Public Health, University of Naples Federico II, 80125 Naples, Italy; federicacrauso@gmail.com (F.C.); giovanni.improta@unina.it (G.I.); 4 Department of Electronics and Telecommunications, Polytechnic University of Turin, 10129 Turin, Italy; luigi.duraccio@polito.it; 5 NexusTLC SRLS, 80010 Quarto, Italy; info@nexus-tlc.com

**Keywords:** wearable systems, wearable sensors, deep learning, LSTM, machine learning, remote health monitoring, vital sign monitoring, telemonitoring, health 4.0

## Abstract

This work addresses the design, development and implementation of a 4.0-based wearable soft transducer for patient-centered vitals telemonitoring. In particular, first, the soft transducer measures hypertension-related vitals (heart rate, oxygen saturation and systolic/diastolic pressure) and sends the data to a remote database (which can be easily consulted both by the patient and the physician). In addition to this, a dedicated deep learning algorithm, based on a Long-Short-Term-Memory Autoencoder, was designed, implemented and tested for providing an alert when the patient’s vitals exceed certain thresholds, which are automatically personalized for the specific patient. Furthermore, a mobile application (*EcO2u*) was developed to manage the entire data flow and facilitate the data fruition; this application also implements an innovative face-detection algorithm that ensures the identity of the patient. The robustness of the proposed soft transducer was validated experimentally on five individuals, who used the system for 30 days. The experimental results demonstrated an accuracy in anomaly detection greater than 93%, with a true positive rate of more than 94%.

## 1. Introduction

The recent COVID-19 pandemic and the associated transition of patient care outside the hospital have boosted the development of systems for the remote monitoring of patient vitals signs [1,2,3], a task that has also been favored by the advancement of wearable technologies [4,5,6,7,8,9] and the Internet of Things (IoT). These two technologies have contributed to the widespread adoption of smart healthcare solutions (soft transducer), deployed either at hospitals or at home [10,11]. In fact, on the one hand, the integration of IoT with wearable devices enables the doctor to remotely monitor the patients’ health. On the other hand, it also allows patients to gain awareness of their health status, which is particularly important when affected by chronic diseases. This approach facilitates an engaging and responsive patient experience, thus improving the *patient’s journey*.

Among chronic diseases, one of the most widespread is certainly hypertension. In fact, the World Health Organization states that one in three adults in the world suffer from hypertension, and this proportion increases with age. Hypertension is frequently referred to as the *silent killer* because it often does not involve disturbing symptoms but still can degenerate suddenly and seriously. Even a moderate increase in blood pressure is associated with reduced life expectancy. In this regard, monitoring patient vitals represents an important aspect of patient care because these signs usually give early information about abnormal physiology. In practice, this can be accomplished by employing soft transducers, which are wearable devices able to acquire and process a large amount of data in real time [12,13]. Indeed, it is crucial for physicians to be able not only to monitor hypertensive patients regularly but also to predict the evolution of this condition.

In the last few years, the processing of data related to patient vitals has been facilitated by the adoption of artificial intelligence (AI), which is one of the most promising enabling technologies of the 4.0 paradigm [14]. In fact, AI represents a strategic tool for supporting clinical decisions and improving disease management [15], thus promoting the correct management, interpretation and use of multiple data collected from the individual patient [16]. The incorporation of AI, and in particular Machine Learning (ML) and Deep Learning (DL), has the potential to improve personalized, patient-centered care medicine, thus strengthening the effectiveness of therapies [17,18,19]. AI can be defined as a technology aimed to provide algorithms that learn from data without being programmed [20,21,22]. ML and DL are a sub-category of AI and refer to data processing oriented to (i) identify and design their relevant characteristics and (ii) perform predictions on the output generated [23]. In Healthcare, the adoption of ML and DL can be considered the best practice in designing decision support systems aimed at predicting patients’ health [24].

Starting from these considerations, this work presents the development of a DL-based soft transducer for the telemonitoring of patient vitals. In addition to the wearable sensors platform for remote monitoring of the vital signs, a DL algorithm based on the Long-Short-Term-Memory (LSTM) Autoencoder was implemented. This choice is driven by the potential of the DL to be able to automatically identify complex features even without having any prior knowledge of the domain. As detailed in the following, the implemented network allows anticipating possible onset or worsening of the disease. The most notable aspect is that, different from the state of the art (see Section 2), the proposed algorithm is trained to identify patient-specific alert thresholds. In fact, the definition of personalized threshold values reduces the occurrence of false positives during normal operating conditions. Finally, to manage data flow and to facilitate data fruition, a dedicated mobile application was developed that also provides an alert to patients and physicians in the case of aggravating conditions. The application also includes a face-recognition feature that allows verifying the patient’s identity. It is important to point out that, while the proposed system was developed and validated in a case study related to healthcare, the obtained results have broader generality and may be declined for other application contexts.

The paper is organized as follows. In Section 2, several approaches similar with the one proposed in this work are discussed, showing strong and weak points. Then, in Section 3, a conceptual description of the proposed soft transducer is provided, and the design of the proposed soft transducer is presented. Section 4 addresses its implementation of the soft transducer, while in Section 5, the experimental results are reported and discussed. Finally, in Section 6, conclusions are drawn and future work is outlined.

## 2. Related Work

As mentioned in the Introduction, AI has been widely used as a solution for predicting patients’ health [24]. For example, in [25], 21 different ML algorithms were applied and compared in the field of hypertension. In [26], a prediction system characterized by the use of an artificial neural network was described to evaluate the risk of hypertension in rural residents over the age of 35 years in a Chinese area. In [27], the authors proposed a hybrid machine learning algorithm of *k-Nearest Neighbor* (k-NN) and *Least-Square Support Vector Machine* (LS-SVM) for predicting future values of monitored vital signs using wearable technologies.In [28], it was found that (i) predictive observation and real-time analysis of values of biomedical signals and (ii) automatic detection of epileptic seizures before onset are beneficial for the development of warning systems for patients as they, once informed that an epilepsy seizure is about to start, can take safety measures in useful time. In [29], a system based on an LSTM network was used in order to monitor vital parameters and ensure an intelligent rehabilitation process. In [30], a novel DL-based anomaly detection approach, called *DeepAnT*, was presented for time series data. It consists of both a time series prediction module and an anomaly detection module. The time series prediction module uses a deep convolution neural network (CNN) to predict the next timestamp on the defined horizon. The expected value is then passed to the anomaly detector module, which is responsible for marking the corresponding timestamp as normal or abnormal. In [31], DL was applied to provide early prediction of type-2 diabetes and hypertension. To perform this analysis, the *Isolation Forest* algorithm was used to detect abnormal data from the data set, while *SMOTETomek* was used to balance the unbalanced data set. Finally, in [32], a forecasting system capable of predicting systolic blood pressure in real time by means of a *Bidirectional Short-Term Memory* (BI-LSTM) algorithm was described.

All these aforementioned works have been demonstrated as a suitable solution to improve real-time patients’ health monitoring. However, a training phase of the algorithms was always required on generalized sets of data. Hence, the resulting alert values are not personalized for the specific patient. As a result, the development of a processing strategy to identify patient-specific features can represent an interesting solution to enhance the patient’s vitals monitoring and the accuracy of the alert provided in case of worsening of health status.

## 3. Design and Overall Architecture

This section addresses the conceptual description of the proposed soft transducer. In particular, the overall architecture and the development of the mobile application are described. Essentially, the proposed soft transducer works as follows.

1. The patient uses wearable sensors to measure the vitals.
2. The measured data are sent to a cloud database and are made available through a mobile application for the patient and the remote physician.
3. The data on the cloud are processed by means of a DL algorithm, which is trained on the basis of preliminary measurements of the patient vitals.
4. If the patient’s vitals exceed a certain threshold, an alert is sent to the physician and to the patient.

**Figure 1:**
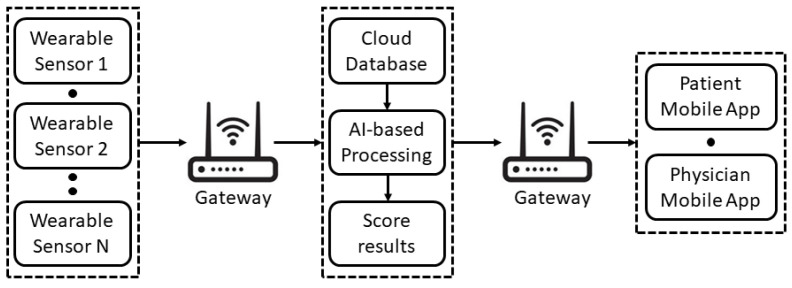
General architecture of the proposed soft transducer.

The overall architecture of the proposed soft transducer is shown in Figure 1. One or more *Wearable Sensors* are used to measure a set of the patient’s vitals. Then, the measurement results are sent to a *Cloud Database*. The obtained data are saved in the database and processed by an *AI Processing* algorithm. The system returns a *Score*, which is sent to the user *Mobile App* along with all the information regarding the data acquired; if the vitals exceed a pre-established threshold, evaluated after training with preliminary measurements of the patient, an alert is sent both to the physician and the patient.

The *mobile application* was developed considering the essential requirements of the healthcare context, including the description of the services offered by the system, the sensor connection, the vital parameter reading, the parameter processing and the activation of emergency alarms. Overall, the application was designed with a six-level structure dedicated to:

1. *Patient registration*;
2. *Vitals measurements*;
3. Management of the patient’s *Medical History*;
4. Remote *Vitals visualization*;
5. *AI processing*;
6. Delivery of the *Score results* to the patient and the physician.

The design of the user interface was carried out taking into account the principles of good system design, as reported in [33]: guaranteeing a minimalist design to prevent cognitive overload, using large and readable icons to facilitate user interaction and, finally, using a clear, concise and intuitive language to help users identify their clinical status.

## 4. Implementation

### 4.1. Wearable Sensing Platform

**Figure 2:**
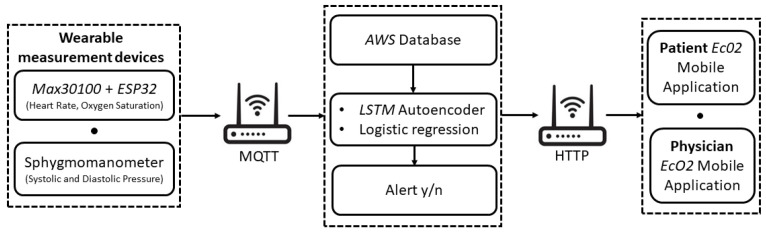
Implementation of the proposed telemonitoring system.

Figure 2 shows the schematization of the wearable sensing platform as implemented in this work.

Heart rate (HR), oxygen saturation (SpO2) and systolic and diastolic pressure (SP, DP) were considered as vitals-to-be-monitored. To this aim, for the monitoring task, the *MAX30100*, a low-cost SpO2 and HR monitor sensor, was used [34].

In order to retrieve the diastolic and sistolic pressure values, the patient is also required to measure their blood pressure through a sphygmomanometer. As detailed in the following section, it is used only once for calibrating the sensor for the successive automated evaluation of the blood pressure starting from HR values.

The wearable sensing platform also includes a low-cost microcontroller with integrated Wi-Fi and dual-mode Bluetooth, namely the *ESP32* [35], allowing the wireless transmission of the measured patient data.

The patient’s vitals are transmitted via Wi-Fi to the database through the MQTT protocol. This database was created and managed in *Node-RED* and works on the *AWS* (Amazon Web Services) cloud platform.

The vital monitoring and real-time anomaly detection is carried out by means of the developed AI-based algorithm. First, a *Multivariate Linear Regression* (MLR) algorithm is used to estimate the value of SP and DP, starting from the HR and SpO2 values coming from the MAX30100 and taking into account the age and the presence of diabetes for each patient. The MLR was chosen since it is one of the most consolidated approaches adopted at the state of the art [36,37,38]. However, other algorithms based on Support Vector Machine, Support Vector Regression [39] and Regression Tree [40] can also be suitably used to estimate systolic and diastolic pressure values. Then, an *LSTM Autoencoder* is implemented to process the entire set of obtained data (HR, SpO2, SP, DP).

**Figure 3:**
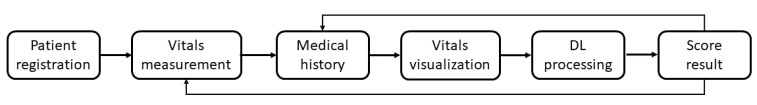
Application level of the proposed telemonitoring system.

Once the measured data are classified, the result is sent in real-time to the mobile application (available to the user and to the physician). In the case of hypertension risk, an alert is also sent to the physician to allow their prompt intervention. As shown in Figure 3, the interactions between the mobile application and Node-RED are managed as HTTP calls.

### 4.2. Mobile Application

The mobile application (which was called *Eco2u*) was developed in Java, and it is compatible with Android (from version 4.4 onward). As previously mentioned, the application is structured in six levels, as shown in Figure 3.

**Figure 4:**
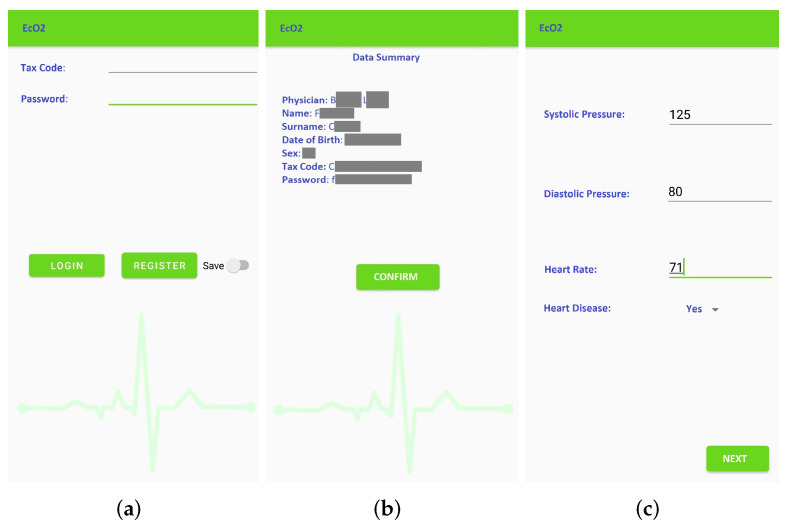
Window of the EcO2u mobile application: Main menu of the application (**a**); Patient registration (sensitive data are hidden) (**b**); Data calibration (**c**).

**Figure 5:**
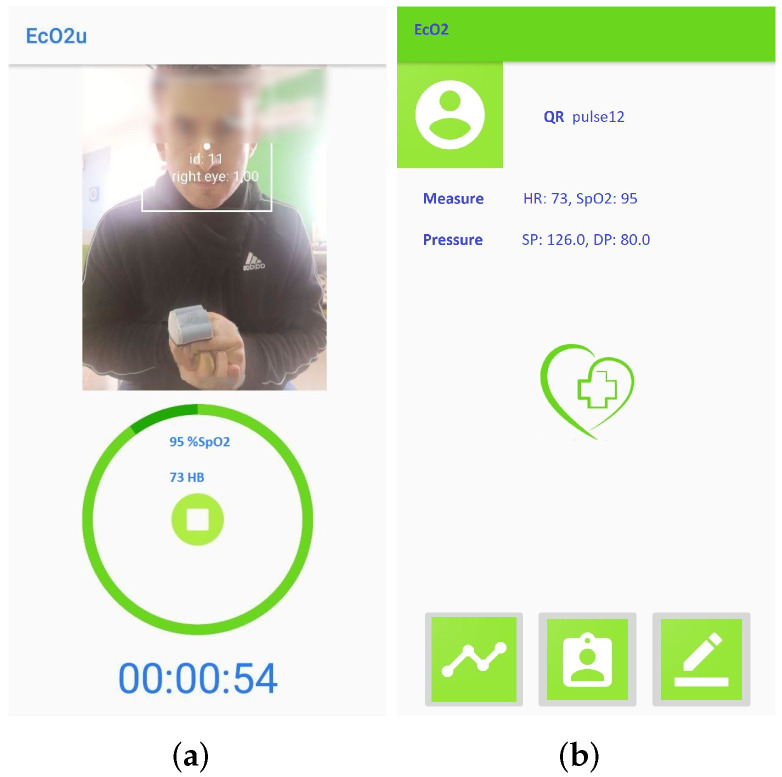
Vitals monitoring with face recognization (**a**); Visualization of vitals after completing the measurement (**b**).

1. *Patient registration*: Figure 4 shows the window for registration and/or log in. During registration, the patient inserts their tax code (which is automatically verified), and the patient is associated to the reference physician. The user also enters additional personal information (such as name, surnames, date of birth). The association to the *wearable measurement devices* is carried out by scanning a QR code generated specifically for a single device. These sensitive data are treated in full compliance with anonymity requirements. In fact, only when an anomaly is detected, the doctor is warned and is able to trace the patient’s identity. Figure 4b shows the window that summarizes the user’s data before they are sent to the *cloud database*, which checks the data and sends a feedback on the correct registration. Once the registration phase is completed, the patient is brought back to the log-in window to make the first log in. Furthermore, at this stage, there is a check with the database to verify that the password and tax code entered are correct.
2. *Vitals measurement*: To allow the successive automated estimation of the systolic and diastolic pressure, a preliminary calibration procedure has to be carried out. In particular, the patient uses a sphygmomanometer to measure the systolic and diastolic pressure values, while the wearable sensing platform sensor is measuring HR and SpO2. Then, the patient manually enters these data in the application (Figure 4c). This phase, which has to be carried out only once, is necessary to identify the parameters that will subsequently allow the soft transducer to estimate the pressure. After the calibration, the patient can start using the soft transducer.
  The mobile application was also endowed with an important identification feature that allows associating (and later verify) the patient’s identity acquired through the smartphone camera, as shown in Figure 5a. Finally, the monitored vitals are displayed to the patient, as shown in Figure 5b.
3. *Management of the patient’s Medical History*: The management of the patient’s medical history is conducted by: (a) showing the daily progress by a graph of the measurements made, (b) reporting the symptoms during the day and (c) indicating any symptoms not present to study a certain correspondence. The cloud database is updated in real-time after each measurement session.
4. *Remote Vitals Visualization*: The physician can access the *EcO2u* mobile application with the *master* credentials to view their patient list; after selecting the specific patient, the physician can display the most recent medical parameters, the graph of past trends and the list of notes, which can be also entered by the patient, in order to see if there is an onset of new symptoms that require a change in therapy (see Figure 5b).
5. *AI processing*: The AI-based processing of the acquired vitals provides a diagnostic tool to detect anomalies in real-time. In particular, as detailed in the following section, a multivariate linear regression is used to estimate the value of SP and DP, starting from the HR and SpO2 values coming from the MAX30100 and taking into account the age and the presence of diabetes for each patient, while a DL algorithm, based on an LSTM Autoencoder, is used to process the entire set of obtained data (HR, SpO2, SP, DP).
6. *Delivery of the Score result*: The Score result is a synthetic quantity that indicates if an anomaly is detected based on the patient’s history and current data. In that case, the physician and the patient are immediately warned about the patient’s condition.

### 4.3. Deep Learning Algorithm for Anomaly Detection

The approach used in this work is semi-supervised learning; in fact, most of the originally available data imported from [41] were not labeled but described the patients normal health conditions. However, with such data, it was possible to train a robust model and evaluate its performance in the validation and test phase using a small amount of labeled data, including normal and abnormal data.

The operating steps of the procedure were the following.

1. *Data Set Creation*: First, the reference data set for the anomaly detection was imported from [41]. The 50 subjects included are 80% men (40) and 20% women (10), with an age range ranging from 26 to 35 years old. Oxygen saturation, heart rate and identification are indicated for each user.
2. *Model Identification and Training*: The second phase consisted of the construction of a normal behavior model using 80% of the imported data set as training data. The identification of this model is necessary to allow the subsequent classification of anomalies when they occur. The model chosen was the LSTM Autoencoder. This structure is characterized by an Encoder, which learns to generate an internal, compressed representation of input data, and a Decoder, which tries to reconstruct the original input on the basis of this internal representation. The Autoencoder was developed with an LSTM neural network. This choice was dictated by the fact that LSTM is the most suitable approach to process data when effects from past events need to be taken into account, differently from CNN, which does not depend on any previous information for prediction since it only uses the current window [42]. The LSTM requires pre-processing the data based on a three-dimensional array that contains the number of observations, the time window and the relevant information. To determine the LSTM architecture, it was considered that the number of layers and the corresponding number of neurons should be high enough to avoid underfitting but, at the same time, should be as low as possible to avoid both overfitting and high computational complexity. Therefore, an input level with 16 nodes, 2 hidden layers with 4 nodes each, and an output level with 16 nodes was chosen. The number of epochs was set to 100 and the batch size to 10. The model was trained by minimizing the reconstruction error, defined as the average absolute difference between the original input and the rebuilt output produced by the decoder.
3. *Alarm Value Identification*: The third phase consisted of the identification of threshold values in order to mark the data as standard or anomalous. These thresholds were determined by the reconstruction errors that the Autoencoder performs in the training phase. An anomaly occurs if the obtained reconstruction error exceeds that threshold; in that case, the corresponding data are marked as anomalous.
4. *Test Validation*: The fourth phase allowed the validation of the threshold identified in the previous step. At this stage, the Autoencoder was provided with labeled data containing two anomalies to be identified. These test data are made up of the remaining 20% of the imported data set. The identification of an anomaly can be seen as a binary classification problem that provides a prediction score as output. The score indicates the certainty of the system that a given observation belongs to the standard class or that there is an anomaly.
  To this aim, the assessment of the obtained model was carried out using three figures of merit: the *Area Under Curve-Receiving Operating Characteristic* (AUC-ROC) curve; the *F1* score; and the *Binary Accuracy*.
  - The ROC curve is plotted following two metrics: *True Positive Rate* (also known as *Sensivity*) and *False Positive Rate*. The True Positive Rate is defined as the number of true positive results divided by the number of all samples that should have been identified as positive. On the other hand, the False Positive Rate is defined as the ratio between the number of negative results wrongly categorized as positive (false positives) and the total number of actual negative results. The ROC curve shows the relationship between the *True Positive Rate* and the *False Positive Rate*. The closer Area Under Curve (AUC) is to 1, the more accurate is the model.
  - The F1 score is calculated based on two metrics: *Precision* and *Recall* (also known as *Sensivity* or *True Positive Rate* and already defined in the previous item). The Precision is defined as the number of true positive results divided by the number of all positive results, including those not identified correctly. The F1 score is obtained as the harmonic mean of the Precision and Recall, and it is a indication of the test’s accuracy.
  - The *Binary Accuracy* represents how well a classification test correctly identifies or excludes a condition. Then, it is defined as the proportion of correct predictions among the total number of cases examined.
5. *Readjustment*: Once the model is validated, it is readjusted after 30 days with further measurements provided by the user. The readjustment aims to identify customized threshold values for the personalized patient care.

## 5. Experimental Results and Discussion

In this Section, the obtained experimental results are presented and discussed. More specifically, a metrological characterization of the soft transducer in terms of validation of (i) the telemonitoring system and (ii) the DL algorithm performance was conducted.

### 5.1. Experimental Validation of the Telemonitoring System

The telemonitoring application *EcO2U* was tested on five volunteers. Firstly, functional testing was carried out to ensure each block worked properly. In particular, during this phase, it was possible to verify (1) the correctness of the *Wearable Sensing Platform*/Subject association within the database and (2) the calls inserted into the database application to correctly use the information.

Then, the correct estimation of systolic and diastolic pressure, obtained by means of the multivariate linear regression algorithm, was verified after inserting the parameters required during the calibration phase. Throughout the measurement, the mobile phone focused on the user and on the sensor in order to validate the procedure. Automatically, the data are sent to the database and made visible to the user. If the measurement result, after appropriate processing, indicates a risk for the patient, then the application itself will manage this alarm by informing the doctor and the patient himself.

For each subject, 30 HR and SP/DP values were recorded. Two different sessions were conducted. Table 1 summarizes the obtained results in terms of mean value and related 1-σ repeatability. The results confirmed the proper functioning of the telemonitoring section of the soft transducer.

### 5.2. Experimental Validation of the Developed DL Algorithm

**Figure 6:**
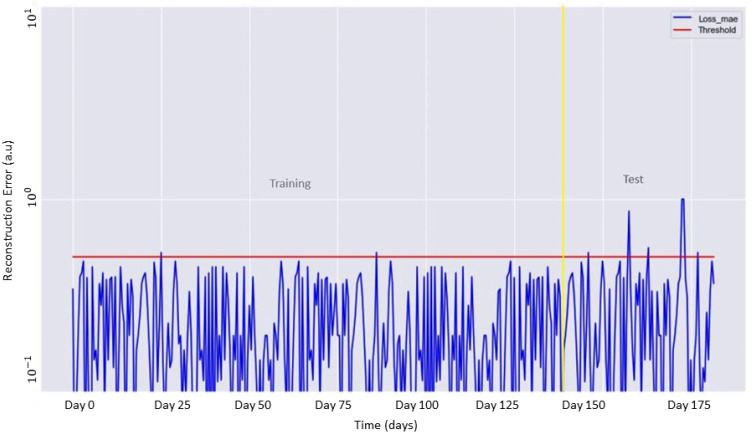
Reconstruction error (blue line) as a function of the training and test data. The identified threshold for the anomaly detection is shown in red.

As mentioned in Section 4.3, the training of the LSTM Autoencoder was carried out for 100 epochs and allowed the identification of the appropriate threshold value. Figure 6 shows the behavior of the Autoencoder on the complete dataset, including the data used for training and testing. These data were indexed day by day. As shown, during the observation period, the score of the reconstruction error occasionally exceeded the threshold value, indicating an anomaly in the measured data.

Table 2 summarizes the results of the binary classification (anomalous/standard data) on the test set in terms of True Positive Rate, False Positive Rate and Precision.

**Figure 7:**
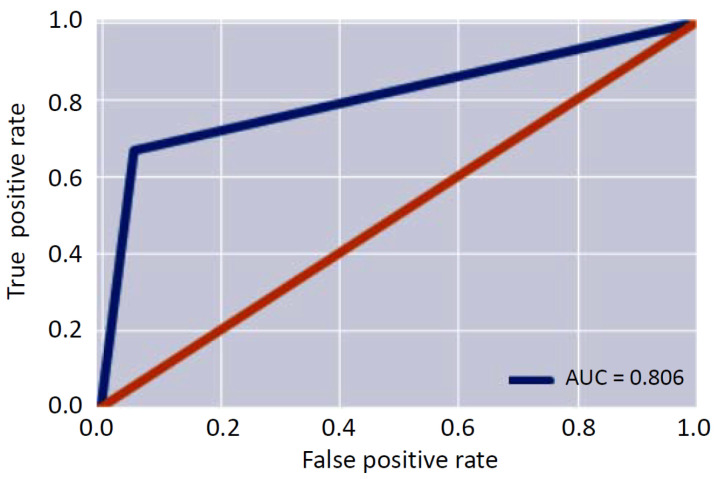
Area under the curve.

Starting from these metrics, the three figures of merit (AUC, F1 score and Binary Accuracy) were evaluated. In particular, the AUC was equal to 0.81, the F1 score was equal to 0.96 and the binary accuracy was equal to 0.93. Figure 7 shows the resulting AUC. The obtained results confirmed the capability of the system to successfully identify the anomalies that can occur during the monitoring phase.

#### Patient-Specific Customization and Validation of The Soft Transducer

The patient-specific customization of the proposed soft transducer (*Readjustment*) was carried out on the five volunteers. The operative steps, conducted separately for each volunteer, were the following:

1. The user employed the soft transducer for 30 days. The acquisition of their vitals (twice a day) also included abnormal values, which were emulated by placing him/her under stress conditions (e.g., a short run).
2. The obtained data set (60 samples) was split into 80% training (48 samples) and 20% test (12 samples). Therefore, the LSTM Autoencoder was re-trained in order to identify the patient specific threshold value

During the test phase, it was observed that the algorithm successfully identified all the labeled anomalies.

After this 30-day phase, further tests were conducted in order to determine the optimal number of days to wait to update the model to maintain adequate performance. The results showed that 15 days is the optimal calibration interval necessary to personalize and update the model. In fact, this choice allowed obtaining a value of AUC equal to 0.831 in the test phase, while after 30 days, this value slightly increased to 0.836.

## 6. Conclusions

In this work, a soft-transducer for remote monitoring of a patient’s health was designed, implemented and experimentally validated. The soft transducer measures the patient’s heart rate, oxygen saturation and systolic/diastolic pressure in real-time and sends the data to a remote database, which can be easily consulted both by the patient and the physician. To endow the soft transducers with predictive features, a DL algorithm (based on LSTM Autoencoder) was developed and implemented: the algorithm provides an alert when the vitals exceed certain thresholds, which are personalized for the specific patient. Furthermore, a dedicated application (named *EcO2u*) was developed (i) to manage the remote collection of the patient vitals and the communication with the physician and (ii) to automatically detect anomalies by means of a patient-personalized, DL-based processing. After validating a public data set, the obtained experimental results on five volunteers showed an accuracy in anomaly detection greater than 93% with a True Positive Rate higher than 94%, thus confirming the robustness of the proposed strategy.

In practical applications, the proposed soft transducer can facilitate the monitoring of patients outside clinical facilities by providing advantages to the hospital in terms of resource management. Moreover, the proposed system manages to improve the quality of the patient’s life by allowing them to stay in their own family environment in contact with family and friends. This benefit is particularly important for children or elderly patients, for whom hospitalization may have severe emotional impact. Finally, it is worth mentioning that, although in this work a healthcare-related case study was considered, the obtained results have broader generality and may be declined for other application scenarios.

Future work will focus on integrating the developed soft transducer into an augmented reality-based interface, which has been proven effective in the medical field [43,44,45,46], in order to further improve patient’s engagement and their *journey*.

## Figures and Tables

**Table 1:** Average values of vitals acquired in two sessions with related 1-σ repeatability.

Subject	HR (Bpm) 1st Session	HR (Bpm) 2nd Session	SP/DP (mmHg) 1st Session	SP/DP (mmHg) 2nd Session
#1	85 ± 3	82 ± 2	112/80 ± 2	112/79 ± 2
#2	71 ± 2	68 ± 2	130/82 ±1	128/82 ± 2
#3	88 ± 4	85 ± 4	125/85 ± 2	124/84 ± 1
#4	75 ± 1	73 ± 2	126/84 ± 2	125/84 ± 1
#5	70 ± 2	67 ± 1	136/82 ± 1	134/82 ± 2

**Table 2:** Results of data classification.

Metric	Result
**True Positive**	68
**False Positive**	1
**False Negative**	4
**True Negative**	2
**True Positive Rate**	0.94
**False Positive Rate**	0.33
**Precision**	0.99
**Area Under Curve**	0.81
**F1 Score**	0.96
**Binary Accuracy**	0.93

## Data Availability

Not applicabile.
